# Fast fitting to low resolution density maps: elucidating large-scale motions of the ribosome

**DOI:** 10.1093/nar/gkt906

**Published:** 2013-09-28

**Authors:** Samuel Coulbourn Flores

**Affiliations:** Computational and Systems Biology Program, Department of Cell and Molecular Biology, Uppsala University, BMC Box 596, 75321 Uppsala, Sweden

## Abstract

Determining the conformational rearrangements of large macromolecules is challenging experimentally and computationally. Case in point is the ribosome; it has been observed by high-resolution crystallography in several states, but many others are known only from low-resolution methods including cryo-electron microscopy. Combining these data into dynamical trajectories that may aid understanding of its largest-scale conformational changes has so far remained out of reach of computational methods. Most existing methods either model all atoms explicitly, resulting in often prohibitive cost, or use approximations that lose interesting structural and dynamical detail. In this work, I introduce Internal Coordinate Flexible Fitting, which uses full atomic forces and flexibility in limited regions of a model, capturing extensive conformational rearrangements at low cost. I use it to turn multiple low-resolution density maps, crystallographic structures and biochemical information into unified all-atoms trajectories of ribosomal translocation. Internal Coordinate Flexible Fitting is three orders of magnitude faster than the most comparable existing method.

## INTRODUCTION

Fitting all-atoms models to low-resolution density maps is a current research topic, and a possible means of predicting conformational change, when said maps are available for alternate conformations (1). In the simplest and most economical approach, rigid-body fitting algorithms (2) can be used to fit domains into density maps one at a time (3). Large complexes have been carefully fitted in this way (4), but the procedure can result in unphysical bonds and steric clashes at the boundary between domains, and it yields no dynamical information. A normal mode expansion (5) can fit a crystallographic structure into a density map, describing a change of conformation. However, normal mode expansions are valid only for small oscillations about equilibrium and cannot describe a system crossing over an energy barrier (6), as occurs in hybridization, completion of translocation and many other ribosomal transitions. The so-called zero modes are not defined, so this approach cannot model overall translation and rotation of one subunit with respect to the others [as occurs e.g. as transfer RNA (tRNA) translocates or 16S rocks with respect to 23S (7)]. A Deformable Elastic Network can overcome these limitations but has only been demonstrated for single protein chains (8,9). Molecular Dynamics Flexible Fitting (MDFF) is a particularly general method, which has been applied to ribosome structures and can even include some restraints from the literature (10,11). However, this requires at least a few dozen processors for the 16S subunit (K. Y. Chan, personal communication), making the method expensive, especially for the much larger, complete ribosome. Multi-Resolution Modeling refers to applying different levels of resolution (coarse to fine) to different spatial or temporal regions of a system (12), a logical approach given the limited capability of coarse-grained approximate methods, and the cost of fine-graining. I present a Multi-Resolution Modeling method called Internal Coordinate Flexible Fitting (ICFF), which complements and addresses the limitations of existing codes. It enables fully atomic forces and flexibility but permits the user to limit these to specified regions, treating the rest of the complex with reduced forces and flexibility. Large complexes like the ribosome can be fitted into low-resolution density maps even where large-scale conformational rearrangements are required. The method is economical enough to easily run on a laptop.

The present work is motivated by the ribosome and, in particular, translocation. Translocation refers to the process in which the ribosome rearranges during the elongation cycle to move tRNAs from the classical state (in which the 16S subunit is in the unrotated state, and the tRNAs are in the P/P and possibly A/A sites) to the hybrid state (in which the 16S is in the rotated state, and the tRNAs have moved to the P/E and A/P hybrid sites), and on to the post-translocation state (in which the 16S returns to the unrotated state, and the tRNAs are in the E/E and P/P sites). I also treat the subsequent state, in which the leaving tRNA attaches to L1 having detached from the E-site.

A considerable number of ribosomal states have been observed crystallographically (13). In particular, for the process of translocation, there are structures of the classical state (14), as well as of an intermediate state between the classical and hybrid states (7). The hybrid state has been observed at low resolution by cryo-electron microscopy (cryo-EM) (15). More recently, Fischer *et al.* (1) reported multiple cryo-EM snapshots along the back-translocation trajectory. However, the trajectories between states are largely unknown, owing to the difficulty of observing them experimentally [e.g. by single molecule methods (16)] or computing them by physical or semi-physical methods (17). This work generates possible trajectories consistent with these density maps.

X-ray crystallography faces a considerable limitation in elucidating transition states. In solution, the molecule must remain in a single conformation long enough to crystallize, favoring a stable state. Diffraction measurements are then taken over a certain time, which is typically long compared with thermal fluctuations (18). Crystallography of transition states relies on unnatural constructs such as mimics of transition state intermediates, (19) non-hydrolyzable or otherwise non-reactive substrate analogs (20), drugs or other molecules that bind transition states (21) and incomplete or otherwise unnatural macromolecular complexes (7). Nuclear magnetic resonance is also done on a large number of molecules (making it harder to track details of motion), monitoring a limited number of atom–atom distances (owing to the problem of separating peaks) and for molecules of limited size (perhaps tens of kDa, for the aforementioned reason) (22). On the upside, methods using a large number of molecules such as the previously mentioned are intrinsically more accurate than single molecule methods.

Single molecule experiments, on the other hand, can observe transient events. Fluorescence resonance energy transfer yields a limited number of point-to-point distances (16). Cryo-EM can also be used to observe single molecules (23), or alternatively many such observations can be clustered to resolve specific substates at higher resolution (1).

On the computational track, methods to predict the dynamics of motion include traditional Molecular Dynamics (24), coarse grained methods (25) and modern multiscale approaches (26). Molecular Dynamics struggles to predict conformational changes *de novo* owing to the long time scales involved; in particular for the ribosome, the most notable studies of such phenomena use biased dynamics (17). Coarse-grained methods are limited in their ability to predict complex large-scale conformational change (6). Much recent success in RNA dynamics has come from multiscale approaches (12), such as that described here.

In this work, I fitted all-atoms structures into multiple density maps along the translocation trajectory (1). Doing this in sequence, with the density maps guiding the motion of the subunits, yielded an all-atoms trajectory not only of the observed states but also of a sterically permitted trajectory connecting them. The trajectory was also guided by known contacts between the tRNA and the 16S head and between the tRNA and its P and E binding sites on 23S.

## MATERIALS AND METHODS

The novel ICFF joins ‘Physics where you want it’, (27) a base-pairing force field (28), threading (27,29) and many other published features (30) in MacroMoleculeBuilder (MMB). MMB is a flexible, general-purpose and internal coordinate modeling suite for protein, RNA and DNA, providing user control over forces, constraints, flexibility and simulation conditions (30).

ICFF is comparable with MDFF in that each atom perceives a force proportional to its atomic number and to the gradient of the electron density at its nuclear position (10); ICFF uses this fitting potential but differs from MDFF in four key ways. First, as the dynamics are computed in internal coordinates, domains can be rigidified, with hinges and other key elements left flexible, resulting in a drastic reduction of degrees of freedom. In contrast, in MDFF, restraints are added to stabilize secondary structure (10), but as the degrees of freedom are not reduced, the effect is to increase rather then decrease computational cost. Second, as in ICFF, the domains are rigid and cannot collapse, no costly interatomic force field is used except in specific regions where clashes might occur (e.g. hinges and interfaces). Third, a wide variety of forces [e.g. base pairing (30,31)] and constraints (e.g. between bound subunits) can be imposed based on additional biochemical and biophysical information. Fourth, rather than using a fixed time step integrator, MMB uses a variable time step, which permits it to take long steps for economy when numerical stability permits and short steps when fast oscillations occur (e.g. when resolving steric clashes) (30). In this section, I validate the novel ICFF and describe other relevant MMB features.

### Density-based force field

ICFF uses a density-based force field, in which Cartesian forces are applied to atomic nuclei, which are proportional to the gradient of the electron density at the nuclear position. This has the following functional form, following MDFF (10):

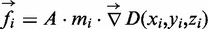

Where *i* is the atom index, 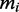 is the atomic number of atom *i*, 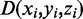 is the electronic density at the nuclear position of atom *i*, and *A* is a user-adjusted scaling factor. *i* counts over all user-selected atoms to be included in the fitting, omitting hydrogens.

### Physics where you want it

**Figure 1.**
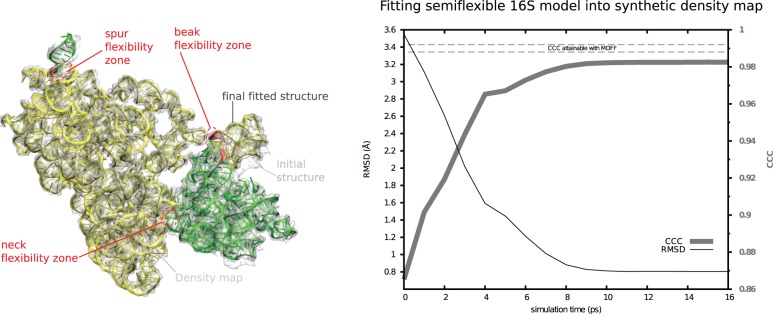
Validation of ICFF with 16S. Left panel: The initial 16S structure (ghost gray cartoons) comes from 2AW7 (33). The model has only 12 flexible residues (in red, surrounded by red dashed ovals). The final fitted structure is shown in opaque green, yellow, green and yellow cartoons, corresponding to spur, body/platform, head and beak rigid domains, respectively. PARM99 forces are active for only 22 residues (not indicated), including the flexible residues and their non-flexible immediate spatial neighbors. This semiflexible model was fitted into a density map (transparent gray) synthesized from 2AVY (33). Right panel: Convergence of fitting. CCC (thick gray trace, values on right axis) is defined in the text. I track CCC as the initial structure is fitted to the density map. Note that final CCC of 0.983 is near the MDFF result (0.988–0.992, gray dashed lines), but ICFF is faster (6 min versus 240 h). As an alternate measure RMSD (thin black trace, values on left axis) is computed against the 2AVY structure. Note that the final RMSD of 0.80 Å is well below the resolution of typical CryoEM density maps. The thus-fitted structure could nonetheless be used as input to MDFF for a short-time final refinement.

**Figure 2.**
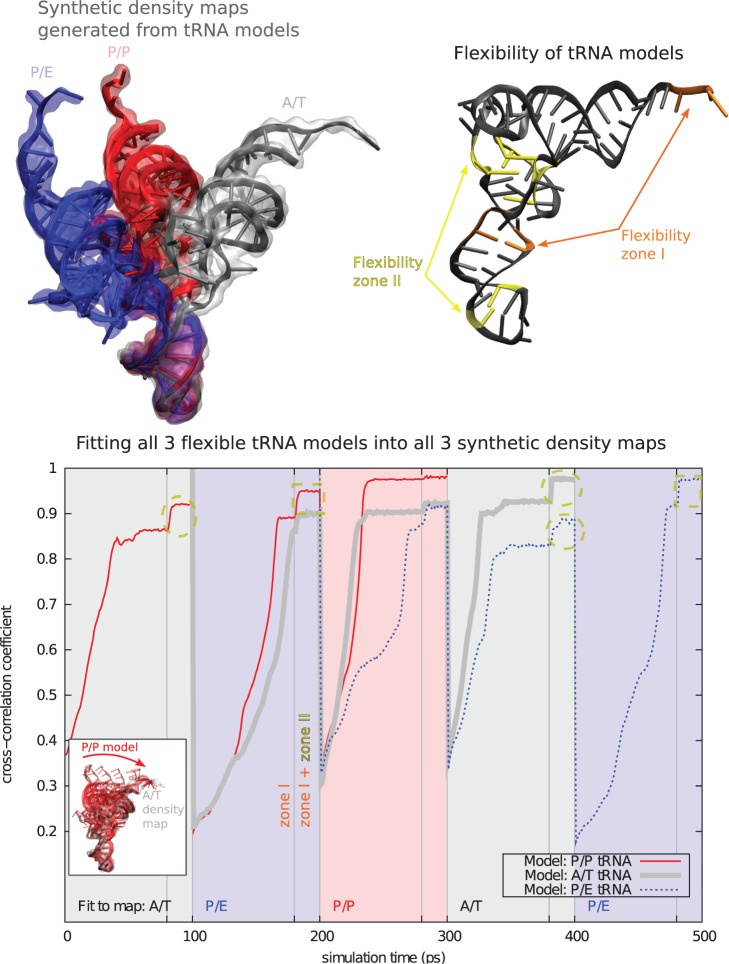
Validation of ICFF with all-versus-all fitting of three semiflexible tRNAs into three synthetic density maps Upper left: Following (34), I aligned P/E [phenylalanine, PDB ID: 4GD1 (34)], P/P [phenylalanine, 4GD2 (34)] and A/T [tryptophan, 2XQD (35)] tRNAs on their anticodons. I then generated one synthetic density map for each structure. Upper right: I used MMB’s ‘Physics where you want it’ to limit flexibility to zone I (middle of anticodon stem and CCA terminus) for initial fitting, then added flexibility zone II (base of anticodon and elbow) for a second finer fitting. The PARM99 force field was active in the flexibility zones, plus nearby non-flexible residues. Lower: Red trace: I did an initial fitting (zone I flexible) of the P/P tRNA model for 80 ps, into the A/T density map (gray shaded region, see also inset). I then did a final fitting (zone I + II flexible) for an additional 20 ps. I then switched to the P/E density map (blue shaded region), and finally to the P/P map (red shading). Similarly, the A/T (gray trace) and P/E (blue trace) models were fitted into all three density maps, for a total of nine fitting runs. Each fitting took an average of 21 CPU-min. Additional increase in CCC as zone II becomes flexible. This is most prominent for fitting to the A/T density map (dashed yellow ovals) reflecting bending at the base of the elbow in the A/T configuration with respect to P/E and P/P. Zone II also increases CCC when the model is fitted to the P/E map after being fitted to A/T (dashed yellow rectangles), as the elbow bending is reversed.

**Figure 3.**
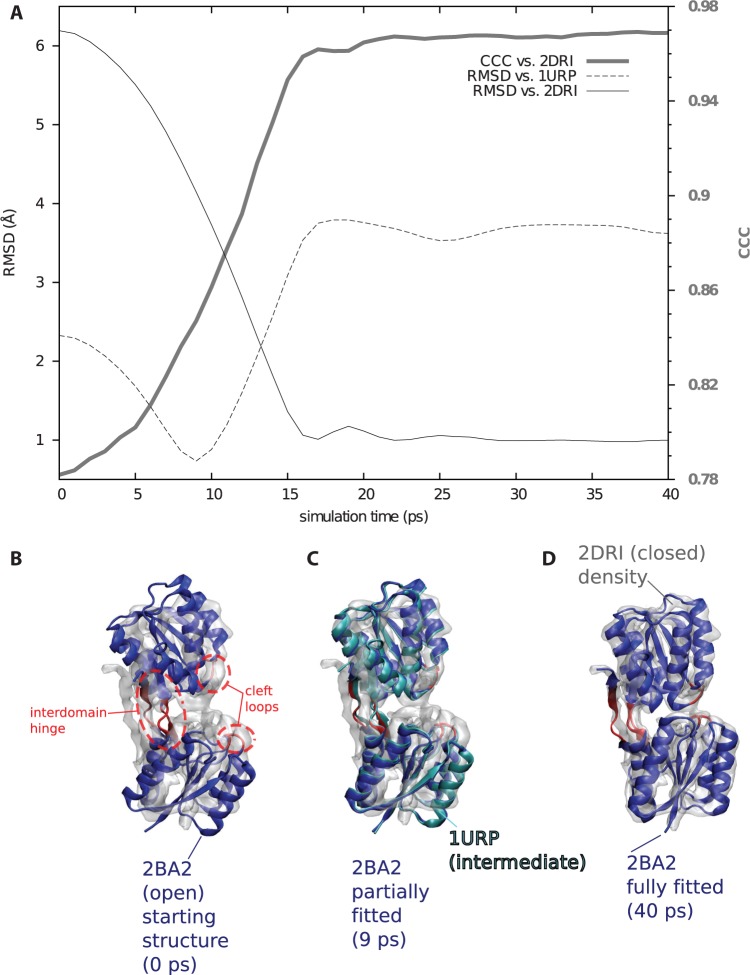
Validation of ICFF by fitting open form (PDB: 1BA2) of RBP to the density map of the closed form (PDB: 2DRI), in the process transiently recapitulating an intermediate form (PDB: 1URP). (**A**) 1BA2 was used as a starting model. Model had an initial RMSD of 6.2 Å with respect to 2DRI; after ICFF fitting to a density map synthesized from 2DRI, this dropped to 0.98 Å (thin continuous black trace, against left axis). The model reached a minimum of 0.73 Å RMSD against 1URP (thin dashed black trace, against left axis), matching the best morphing method of (36). CCC of the model against the 5 Å density map synthesized from 2DRI (thick gray trace) was initially 0.78, increasing to 0.97 on completion of fitting. (**B**) 2BA2 starting model. Red residues (circled in dashed ovals): in model, flexible hinges allow interdomain movement. Flexible loops in the cleft help recover atomistic interactions in the closed form. A physics zone is active in a 10 Å neighborhood around the flexible residues (not shown). Blue: remaining (rigid) residues. Ghost gray: 2DRI density map. Same coloring used in (**C** and **D**). (C) The fitted model at its closest approach to 1URP (cyan), demonstrating ICFF’s potential for recapitulating intermediates. (D) Converged model.

In prior work, we introduced ‘Physics where you want it’ (27), which turns on the PARM99 (32) force field in limited user-specified physics zones. In the 16S (Figure 1), tRNA (Figure 2), and ribose binding protein (RBP) (Figure 3) validation examples, as well as at key points in the translocation trajectory, this feature is used to maintain physically reasonable interatomic distances.

### Internal coordinate morphing

Morphing refers to the generation of a sterically feasible trajectory connecting two macromolecules of known structure (37). However, most existing methods have only a limited ability to deal with highly non-linear trajectories, and further are not economical for large complexes (37). In prior work (27), we showed how threading can be done by using springs to pull a model of unknown structure [instantiated as a flexible, extended chain with collision-detecting soft spheres (30) on all atoms] onto a template of known structure. MMB morphing is similar, differing from our threading technique (27) only in that the model is initially a folded structure, and has flexibility only at hinge points. The springs then align the model onto the template following a clash-free trajectory as permitted by user-specified flexibility.

### Convergence by cross-correlation coefficient

Following (11), I measured convergence of the trajectory versus the respective density maps using MDFF’s cross-correlation coefficient (‘CCC’) function (though not any flexible fitting functions). This first computes a simulated density map ‘S’ from the atomic coordinates by projecting atomic numbers onto a grid and then applying a Gaussian filter (11). It then computes the Pearson’s correlation coefficient between S and the experimental density map ‘E’:

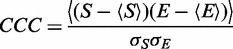

Here map ‘E’ is PRE1, PRE2, and so forth as appropriate for each stage of the fitting procedure. I computed CCC for the validation case (16S 2AW7–2AVY) as well as for the entire translocation trajectory. In the validation case, the initial coordinates came from 2AW7, whereas the map was generated from the coordinates of 2AVY. In the translocation application, the maps were provided by the authors of (1).

### Validating ICFF with 16S

I compared ICFF performance with MDFF as follows. The *Escherichia coli* 16S subunit from PDB structure 2AW7 (33) was the starting model (11). 2AW7 is from a crystal whose asymmetric unit contains a second structure of the ribosome (2AVY) in a different configuration of the head domain (33). I generated a simulated density map from the atomic coordinates of the 16S subunit from 2AVY (33) as above, using a 5 Å Gaussian filter (11).

I first rigidly fitted the starting structure from 2AW7 into the synthetic density map using COLORES, (2) yielding a CCC of 0.865. Using ‘Physics where you want it’, I then created flexibility zones in known hinges (38,39) at the neck, base of the beak and base of the spur (helix h6) (Figure 1); the physics zone (27) included these residues and their immediate spatial neighbors. ICFF converged in ∼6 Central Processing Unit (CPU) min. The final CCC was 0.983, only slightly lower than that obtained by MDFF [0.988–0.992, dependent on grid density (10), see Figure 1]; however, MDFF had an estimated computational cost of 5 h on 48 processors (∼240 CPU-h) (K. Y. Chan, personal communication).

### Validating ICFF with tRNA

As a second validation, I semiflexibly fitted each of three different tRNA structures (P/E, P/P and A/T) into synthetic density maps generated from the same three structures, for a total of nine fitting runs. I defined two flexibility zones. Zone I consisted of the unstructured CCA end and a hinge in the anticodon stem following (40). Significant bending at the elbow region was necessary to fit P/E and P/E models into the A/T density. This was provided by zone II, consisting of a hinge at the elbow and near the anticodon loop (Figure 2). In each fitting, I first did a coarse fitting with only flexibility zone I active, followed by a finer fitting with zones I and II active. In this way, I was able to fit all three models into all three density maps, with CCC ranging from 0.89 to 0.98 (Figure 2 and Supplementary Movie). The approach is economical, costing ∼21 CPU-min for each of nine fitting runs.

### Validating ICFF with RBP

As a final validation, I used an open form of RBP (PDB ID: 2BA2) as a starting model and fitted to a closed form (PDB ID: 2DRI). The interdomain hinges of RBP are annotated in the Hinge Atlas Gold (41) and can also be predicted computationally (41,42). They have been used as the basis for predicting ligand binding motions (26). In addition to the interdomain hinge residues, I also flexibilized two dynamic loops that change conformation in the closed form (Figure 3). The structure of an intermediate form of RBP is available (PDB ID: 1URP), providing a convenient means of determining whether a computed trajectory can recapitulate an intermediate (36). I turned on ‘Physics where you want it’ in a 10 Å region about the flexible residues. Not only was the fitting very high in quality (0.97 CCC, 0.98 Å RMSD versus 2DRI) but it also recapitulated 1URP with 0.73 Å RMSD (Figure 3), matching the best morphing method benchmarked in (36).

### Fitting to Fischer density maps

**Figure 4.**
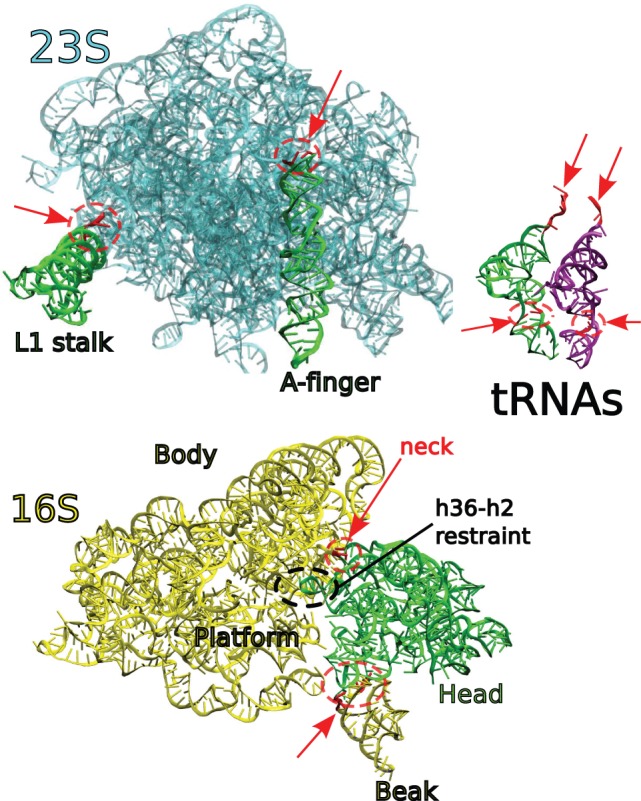
Flexibility of ribosomal RNA Flexible residues in RNA are indicated in red, with red arrows. Different colors highlight specific domains (including 23S L1 stalk, A-finger and 16S Body/Platform, Head, and Beak). Collision-detecting spheres (30) and ‘Physics where you want it’ (27) zones (not shown) prevent clashes between moving units. Note the h36–h2 restraint, which together with the neck hinge restricts head motion.

**Figure 5.**
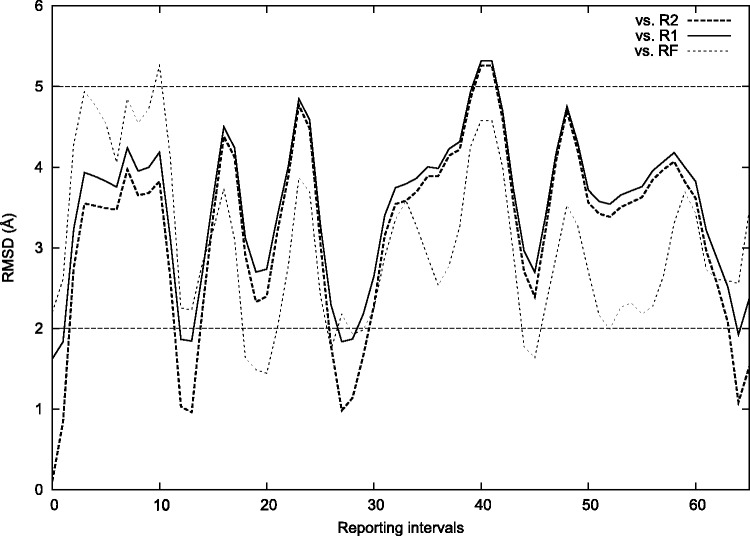
Validating 16S neck flexibility I flexibilized the neck, but not the base of the beak (shown in Figure 4). 16S has an important restraint between a GNRA loop on h36 on the head and a receptor on h2 on the body (also in Figure 4). I enforced this using base-pairing interactions. This significantly reduces the conformational range of the head to lie mostly about a single rotational axis. The head would otherwise have a wide range of rotational motion on three axes. Thermally exploring the accessible configurations resulted in repeatedly recapitulating the classical (R1), (7) intermediate (R2) (7) and hybrid (RF) (43) configurations of the head, within 2 Å RMSD (solid, dashed and dotted traces). RMSD is computed for the head domain after aligning the body/platform. Gate widths of ∼20 Å occurred transiently. Computer time was <1 min, thus the internal coordinate sampling approach is economical.

Here I describe the method for fitting the ribosome to the multiple back-translocation density maps (1). I left most of the ribosome rigid for economy and flexibilized only key hinge points (Figure 4) (43). For 16S, these include the neck (7,39), connecting head and body domains, and the base of the beak (39). For 23S, I flexibilized the base of the L1 stalk (37) and of the A-finger (Table 1) (44). I applied a restraint representing the tetraloop-receptor interaction between h36 and h2 (Figure 5). I did not explicitly account for platform flexibility (45), as the displacements are smaller and did not increase gate width (33).

**Table 1.** Flexible residues

Subunit	Residues	Description
16S	926, 1391	Neck
16S	989, 990, 1043, 1044	Base of beak
23S	2014, 2015, 2090	Base of L1 stalk
23S	848, 928	Base of A-finger (H38)
tRNAs	72–76	Acceptor terminus
tRNAs	26, 44	Anticodon stem (during hybridization)

Residue numbers follow the numbering in the *T. thermophilus* ribosome structures used as initial models (PDB ID: 2J02, 2J03). Note that the many thousands of degrees of freedom corresponding to all atoms have thus been reduced to fewer than 200. This internal coordinate scheme was used in all stages of the fitting except where noted.

**Figure 6.**
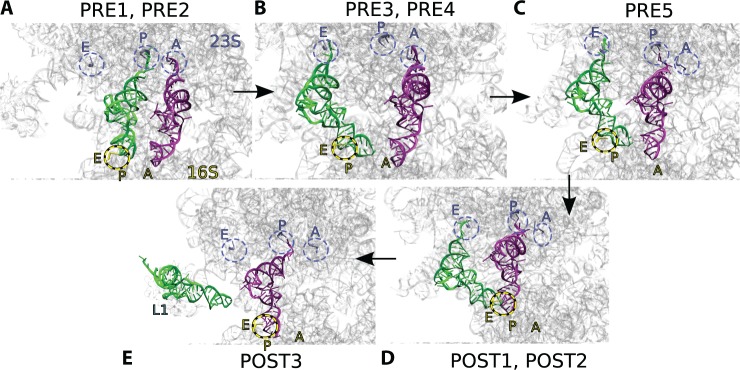
Additional forces. As a complement to the low-resolution density maps, specific interactions determine fine details of structure. Canonical and non-canonical base pairing (30) and stacking interactions are enforced between the tRNAs and the 23S A, P and E sites (gunmetal Cartoon residues, gunmetal dashed ellipses) according to the state (PRE1 to POST3) along the translocation trajectory. The Shoji interaction binds tRNA to the 16S P-site (yellow cartoon residue, yellow dashed ellipses). **A**, **B** and **D** show states that differ slightly in conformation but have the same base-pairing interactions. (**E**) Motion of exiting tRNA from E site (POST2) to L1-bound (POST3) was assisted with an internal coordinate morph.

ICFF then guided the thus-prepared ribosome into successive density maps. However, the density maps were not the only source of structural information. There is an important interaction between tRNA and the 16S head (46), which is maintained throughout hybridization (43) and is broken in POST1. The acceptor terminus of the exiting tRNA makes Watson–Crick interactions with the 23S P-site at stages PRE1 and PRE2. The same terminus makes a stacking interaction with the 23S E-site in states PRE4 to POST2. The entering tRNA acceptor terminus makes Watson–Crick and Sugar–Edge interactions (47) with the 23S A-site up to stage PRE4. The same terminus then makes Watson–Crick interactions with the P-site, which are enforced at stage PRE5. These interactions were represented with base pairing forces (30,31) at the appropriate stages (Figure 6). During some transitions, close contacts initially occurred; as one means of avoiding these, I used ‘Physics where you want it’ in the potential clash regions. This is summarized in Table 2.

**Table 2.** Additional forces

Reporting interval	Dens. map	Base interactions	Comments
1–3	PRE 1	16S:1338, tRNA:41	Shoji interaction
		23S:2436, 2466, entering-tRNA: 75, 76	Entering-tRNA in A-site
		23S:2134, 2135, exiting-tRNA: 78, 79	Exiting-tRNA in P-site
4–78	PRE 2	23S:2436, 2466, entering-tRNA: 75, 76	Entering-tRNA in A-site
		23S:2134, 2135, exiting-tRNA: 78, 79	Exiting-tRNA in P-site
		16S:1338, exiting-tRNA:41	Shoji interaction
79–161	PRE3	16S:1338, tRNA:41	Shoji interaction
		23S:2436, 2466, entering-tRNA: 75, 76	Entering-tRNA in A-site
		23S:2304, exiting-tRNA:80 Stacking	Exiting-tRNA in E-site
162–202	PRE 4	16S:1338, tRNA:41 Sugar Edge	Shoji interaction
		23S:2436, 2466, entering-tRNA: 75, 76	Entering-tRNA in A-site
		23S:2304, exiting-tRNA:80 Stacking	Exiting-tRNA in E-site
203–217	PRE 5	16S:1338, tRNA:41Sugar Edge	Shoji interaction
		23S: 2134, 2135, entering-tRNA:75, 76	Entering-tRNA in P-site
		23S:2304, exiting-tRNA:80 Stacking	Exiting-tRNA in E-site
218–288	POST1	23S: 2134, 2135, entering-tRNA:75, 76	Exiting-tRNA in E-site
		23S:2304, exiting-tRNA:80 Stacking	‘Physics where you want it’ turned on to Establish and maintain reasonable tRNA-16S distances.
		‘Physics where you want it’: 16S:676–679, 1319–1321 tRNA:45–48 16S:1338, exiting-tRNA:41	Shoji interaction
289–314	POST2	23S: 2134,2135, entering-tRNA:75,76	Exiting-tRNA in E-site
		23S:2304, exiting-tRNA:80 Stacking	‘Physics where you want it’ turned on to establish and maintain reasonable tRNA-16S distances.
		‘Physics where you want it’: 16S:676-679, 1319-1321 tRNA:45-48 16S:1338, exiting-tRNA:41	Shoji interaction
315–398	POST3		Guide tRNA to ejected position in density map using positional force.
			Release tRNA forces, relax 16S and 23S into density map.

‘Reporting interval’ is an index that counts over successive structures in the provided trajectory (Supplementary Materials).

Generally, I used ICFF to fit the semiflexible ribosome into each of the eight successive CryoEM density maps. At some stages, the base-pairing forces served to restrain motion and resolve fine structural details of the interactions between tRNA and 16 and 23S. At certain stages, Coulomb and van der Waals interactions were turned on for limited residues on the tRNA, 16 and 23S, where physically reasonable interatomic distances needed to be maintained. Collision-detecting spheres were placed in regions of interaction, e.g. between tRNA and its binding sites on 16 and 23S, as needed to prevent clashes. For POST3, the distance to be traversed by tRNA was large, so I did an internal coordinate morph to its target location, before completing the fitting with ICFF. Morphing forces also sped up initial fitting to PRE2 and PRE3.

**Figure 7.**
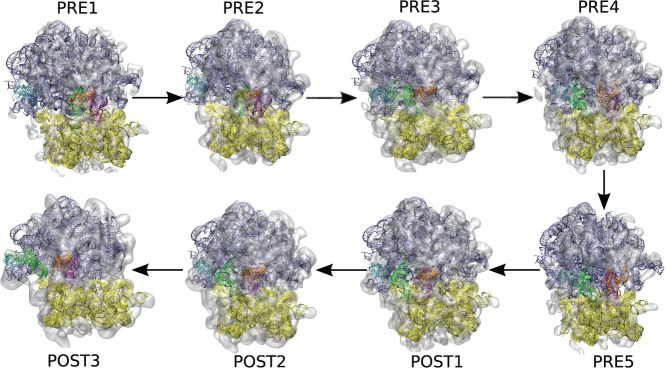
Intermediate 70S ribosome structures fitted to low-resolution density maps using ICFF. 23S (blue), 16S (yellow), tRNAs (green, magenta), L5 (orange) and L1 (cyan) models fitted to CryoEM density maps (ghost gray). Initial coordinates were from PDB entries 2J02, 2J03 (14).

**Figure 8.**
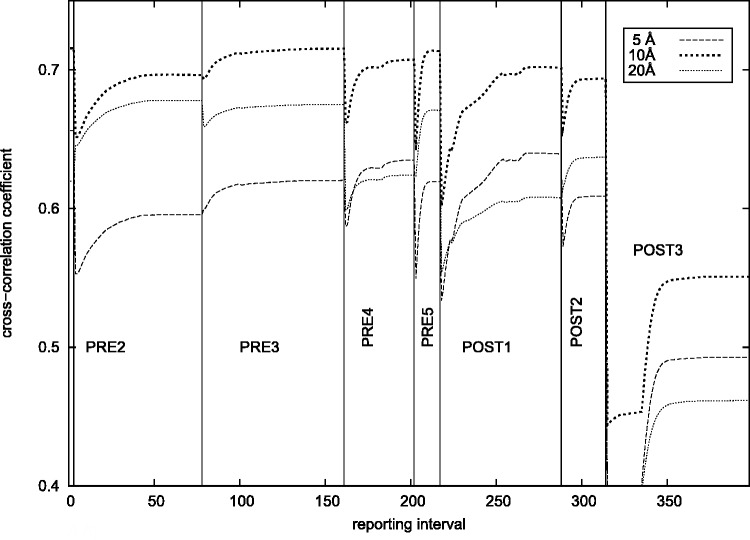
Convergence of fitting to density maps along translocation trajectory. CCC is the inner product of the simulated versus experimental atomic density (see text). Labels (PRE2, PRE3, etc.) indicate density map against which fitting was done and CCC calculated; note CCC accordingly changes abruptly when the density maps are changed (thin vertical lines). Each trace corresponds to a given resolution of the simulated density maps (5, 10, 20 Å). The experimental maps range from ∼9 to 20 Å resolution. POST1 and POST3 fitting were done in stages. ‘reporting interval’ counts over the successive structures in the trajectory.

Using ICFF, base interactions, ‘Physics where you want it’, and other MMB features, I generated a trajectory of motion of the *Thermus thermophilus* ribosome, as it transited through the PRE1, PRE2, PRE3, PRE4, PRE5, POST1, POST2 and POST3 structures (Figure 7) (1). I monitored the convergence by computing the CCC and verified that the latter had plateaued at each stage (Figure 8). The model included 16S, 23S, two tRNAs and proteins L1 and L5. The latter two subunits were featured in (1) owing to their direct interactions with the tRNAs; the influence of other proteins is less evident at this resolution.

## DISCUSSION

I presented a new MMB feature called ICFF, which builds on MDFF. My internal coordinate treatment, however, reduces degrees of freedom for savings in computer time. Rigidifying domains also removes much of the need for an all-atoms physical force field, which drives the cost of MDFF. My 16S validation example converged three orders of magnitude faster (6 min versus ∼240 CPU-h) in ICFF than in MDFF, with nearly the same accuracy. I also did an all-versus-all fitting of three very different tRNA structures into density maps synthesized from them; I used a simplified model of flexibility, attaining accuracy and economy. A similar validation using RBP recapitulated an intermediate form within 0.73 Å RMSD, suggesting the method is useful for finding structural intermediate conformations. ICFF is so economical that the entire ribosomal trajectory was done on a single laptop processor, an impossible task with MDFF. As a further methodological advance, I showed how MMB can be used to generate highly non-linear user-controllable morphs of a large complex using minimal computer time. The tool promises wide applicability to modeling complex conformational change of large macromolecular complexes.

To my knowledge, this is the largest-scale all-atoms trajectory of the 70S ribosome created to date, in terms of the extent of the conformational change. I have fitted 16S, 23S, L1, L5 and tRNA subunits to eight low-resolution density maps (PRE1, 2, 3, 4, 5 and POST1, 2, 3), well explaining the observed density in seven of these. I created a kinematic trajectory, which shows how these states may be connected. The results highlight several key ribosomal features, such as steric barriers in completion of translocation (48). The trajectory should be useful in rational design of fluorescence resonance energy transfer (17) and other experiments.

## AVAILABILITY

The trajectory, structural snapshots corresponding to PRE1, PRE2, PRE3, PRE4, PRE5, POST1, POST2, and POST3, and movies of elongation and tRNA fitting are posted at https://simtk.org/home/ribosome-icf. The MMB input files are available on request. MMB is available for download from https://simtk.org/home/rnatoolbox.

## SUPPLEMENTARY DATA

Supplementary Data are available at NAR Online.

## FUNDING

Faculty of Science and Technology at Uppsala University, eSSENCE (essenceofescience.se), the Uppsala RNA Research Center (funded by the Swedish Research Council), and The Swedish Foundation for International Cooperation in Research and Higher Education (STINT). Funding for open access charge: Uppsala University.

*Conflict of interest statement*. None declared.

## Supplementary Material

Supplementary Data
