# Supplementary material for: Fast fitting to low resolution density maps: elucidating large-scale motions of the ribosome
Source: Nucleic Acids Res. 2013 Sep 28;42(2):e9. doi: 10.1093/nar/gkt906 (PMC3902909; doi:10.1093/nar/gkt906)
Supplement: Supplementary Data [file supp_42_2_e9__index.html]

Fast fitting to low resolution density maps: elucidating large-scale motions of the ribosome — Supplementary Data 

# Fast fitting to low resolution density maps: elucidating large-scale motions of the ribosome

## Supplementary Data

files

**Files in this Data Supplement:**

- Supplementary Data - pdf file
- Supplementary Data - mpg file
- Supplementary Data - mpg file
- Supplementary Data - docx file
