# Supplementary figures and images for: Fast fitting to low resolution density maps: elucidating large-scale motions of the ribosome
Source: Nucleic Acids Res. 2013 Sep 28;42(2):e9. doi: 10.1093/nar/gkt906 (PMC3902909; doi:10.1093/nar/gkt906)

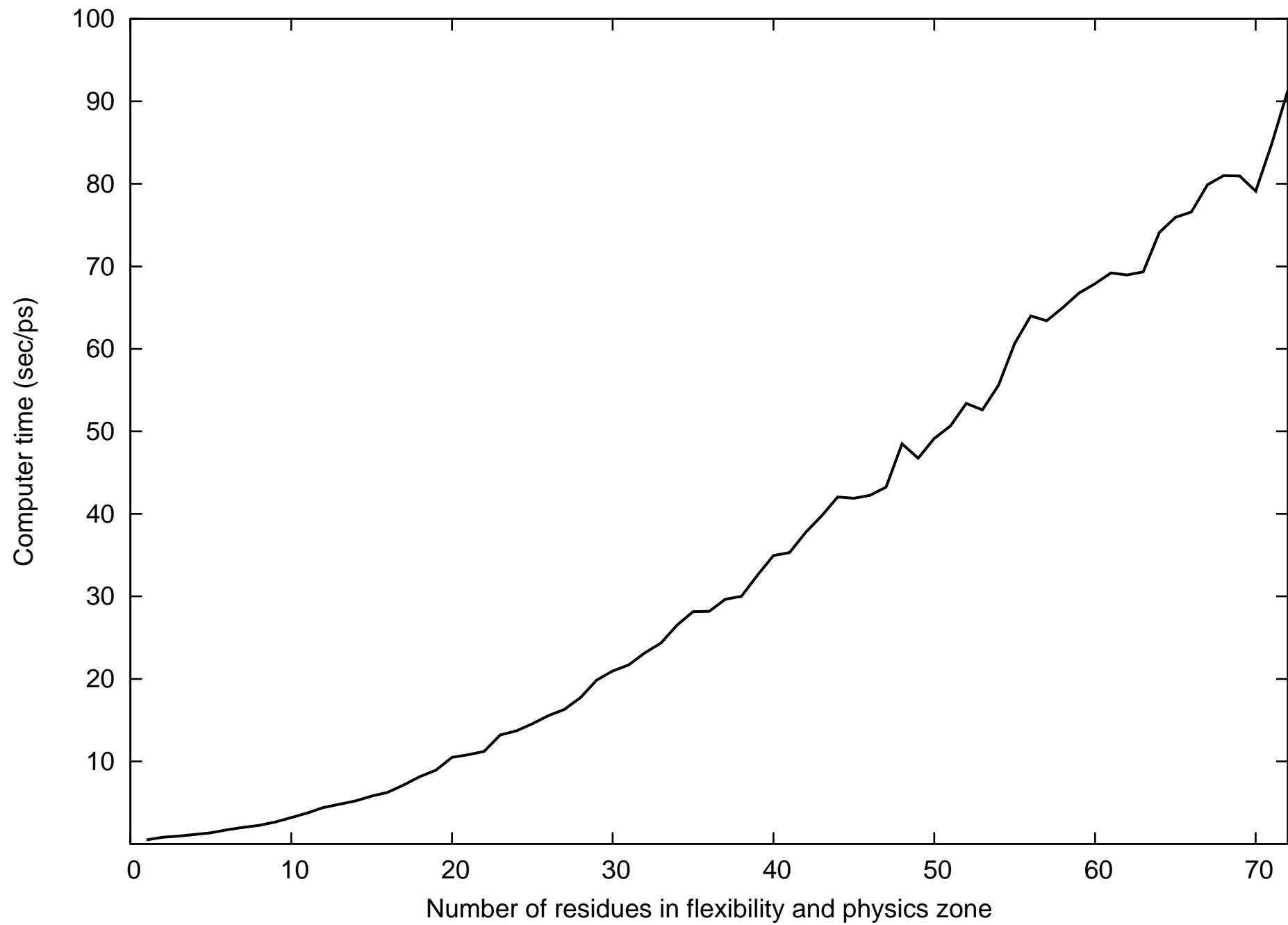

Supplement: Supplementary Data [file supp_gkt906_nar-02483-met-g-2013-File012.pdf]
