# Supplementary material for: Fast fitting to low resolution density maps: elucidating large-scale motions of the ribosome
Source: Nucleic Acids Res. 2013 Sep 28;42(2):e9. doi: 10.1093/nar/gkt906 (PMC3902909; doi:10.1093/nar/gkt906)
Supplement: Supplementary Data [file supp_gkt906_nar-02483-met-g-2013-File013.docx]

Supplementary Figure

Computational cost as number of residues in the flexibility and physics zone increases. I fitted the P/E tRNA into its own synthetic density map, following the procedure described in the text and Figure 2. However I altered the flexibility and physics zones, such that both consist of a stretch of residues ranging form 1 to *x*, where *x* systematically increases from 1 to 72. The computational cost per ps is reported, averaged over 20 ps per run. Cost is thus a relatively smooth function of *x.* Cost of updating internal coordinates *per time step* is a linear function of number of degrees of freedom (50) but we used a variable time step integrator, and thus compute time is a complex function of degrees of freedom, geometry, roughness of potential surface, and constraints. Further, evaluating the PARM99 force field adds a cost that is quadratic in number of atoms. Excessive flexibility is not recommended as this risks destabilizing domains and overfitting.
